# A dedicated paediatric [^18^F]FDG PET/CT dosage regimen

**DOI:** 10.1186/s13550-021-00812-8

**Published:** 2021-07-19

**Authors:** Christina P. W. Cox, Daniëlle M. E. van Assema, Frederik A. Verburg, Tessa Brabander, Mark Konijnenberg, Marcel Segbers

**Affiliations:** Department of Radiology & Nuclear Medicine, Erasmus Medical Center, Postbus, 2040 3000 CA Rotterdam, The Netherlands

**Keywords:** Image quality, PET, [^18^F]FDG activity, Dose optimization, Patient size, Body weight

## Abstract

**Background:**

The role of 2-[^18^F]fluoro-2-deoxy-D-glucose ([^18^F]FDG) positron emission tomography/computed tomography (PET/CT) in children is still expanding. Dedicated paediatric dosage regimens are needed to keep the radiation dose as low as reasonably achievable and reduce the risk of radiation-induced carcinogenesis. The aim of this study is to investigate the relation between patient-dependent parameters and [^18^F]FDG PET image quality in order to propose a dedicated paediatric dose regimen.

**Methods:**

In this retrospective analysis, 102 children and 85 adults were included that underwent a diagnostic [^18^F]FDG PET/CT scan. The image quality of the PET scans was measured by the signal-to-noise ratio (SNR) in the liver. The SNR liver was normalized (SNRnorm) for administered activity and acquisition time to apply curve fitting with body weight, body length, body mass index, body weight/body length and body surface area. Curve fitting was performed with two power fits, a nonlinear two-parameter model α p^−d^ and a linear single-parameter model α p^−0.5^. The fit parameters of the preferred model were combined with a user preferred SNR to obtain at least moderate or good image quality for the dosage regimen proposal.

**Results:**

Body weight demonstrated the highest coefficient of determination for the nonlinear (R^2^ = 0.81) and linear (R^2^ = 0.80) models. The nonlinear model was preferred by the Akaike’s corrected information criterion. We decided to use a SNR of 6.5, based on the expert opinion of three nuclear medicine physicians. Comparison with the quadratic adult protocol confirmed the need for different dosage regimens for both patient groups. In this study, the amount of administered activity can be considerably reduced in comparison with the current paediatric guidelines.

**Conclusion:**

Body weight has the strongest relation with [^18^F]FDG PET image quality in children. The proposed nonlinear dosage regimen based on body mass will provide a constant and clinical sufficient image quality with a significant reduction of the effective dose compared to the current guidelines. A dedicated paediatric dosage regimen is necessary, as a universal dosing regimen for paediatric and adult is not feasible.

## Background

Currently, the role of 2-[^18^F]fluoro-2-deoxy-D-glucose ([^18^F]FDG) positron emission tomography/computed tomography (PET/CT) imaging is still expanding in the diagnosis and follow-up of paediatric oncologic, infectious and inflammatory diseases [1, 2]. This expansion brings a point of concern, since both the CT and the [^18^F]FDG expose patients to ionizing radiation, which can cause radiation-induced effects later in life [3]. The risk of radiation-induced carcinogenesis is higher in children, as they have a longer post-radiation exposure life expectancy compared to adults [3]. To reduce this risk, the radiation dose of paediatric PET/CT scans should be as low as reasonably achievable (ALARA) [4] with acceptable image quality and within a reasonable acquisition time. Therefore, optimization and harmonization of paediatric PET/CT imaging protocols are essential [5].

For performing paediatric nuclear medicine procedures, both the Society of Nuclear Medicine and Molecular Imaging (SNMMI) and European Association of Nuclear Medicine (EANM) recommend the EANM paediatric dosage card (version 5.7.2016) [6, 7] or the 2016 North American Consensus guidelines (NACG) [7, 8]. Both these guidelines have, however, several shortcomings as they are derived from adult-based protocols [9–12], and both focus on radiation dose without taking image quality into account [9–13]. Moreover, the EANM paediatric dosage card recommends even higher administered activities per kilogram than the adult 2015 EANM [^18^F]FDG guidelines [14]. The optimized adult guidelines recommend a quadratic dosage regimen based on body mass to obtain sufficient constant image quality, which was reported by a study of de Groot et al. [15] using signal-to-noise ratio as a surrogate for image quality. This study investigated the relationship between a patient-dependent parameter, for example, body mass, BMI, LBM, fat mass (defined by body mass minus the lean body mass) and body mass per body/length and the administered [^18^F]FDG. Another option is to use a linear dosage regimen < 75 kg and a quadratic dosage regimen > 75 kg to compensate for the lower signal-to-noise ratio due to excessive attenuation in heavier patients. Optimizing image quality in these patients was investigated by several studies [16–20].

In contrast to adult dose regimens, only a few studies have been published on optimizing administered activities in children. Accorsi et al. [10] found that weight was the best patient-dependent indicator for the administered activity necessary to obtain constant sufficient image quality. A pilot study of van Gent et al.[21] focussed on body weight showed that a linear relationship between body weight and administered activity results in a constant image quality. Other studies have reduced the administered [^18^F]FDG activity per kilogram of body weight based on simulations of PET low-dose scans by reduction of count rates [11, 22, 23].

The Paediatric Dosage Harmonization Working Group and the 2020 paediatric guideline stated that more data are needed for evidence-based optimisation of the current guidelines [7, 24]. This can be achieved by dedicated paediatric studies, based on the methods used for the adult guidelines, for different PET systems and reconstruction methods to provide the needed data for updating the paediatric guidelines. The aim of this study, therefore, is to investigate the relation between patient-dependent parameters and [^18^F]FDG PET image quality and to propose a dedicated paediatric dose regimen that provides a constant and clinical sufficient image quality.

## Methods

### Patients

This retrospective study consecutively included 102 children (54 boys and 48 girls; mean age 12.5 ± 4.6 years; range 0.5–17.9 years) that underwent a diagnostic [^18^F]FDG PET/CT scan at the Erasmus Medical Centre between January 2017 and July 2020. Inclusion criteria were: preparation according to local protocol; serum glucose < 7.0 mmol/L; administered activity ± 10% of the recommended activity; PET acquisition time 60 ± 5 min post-injection; and when disease was present no signs of extensive liver involvement on PET images. The same criteria were used to select 85 adult patients (26 male and 59 female; mean age 57.0 ± 16.5 years; range 22.0–83.0) with as much as possible the same body weight distribution to compare with the paediatric results. The study was approved by the Medical Ethical Committee of the Erasmus Medical Centre (MEC-2021–0078), and procedures were in accordance with the Declaration of Helsinki of 1964, as revised in 2013.

### Patient-dependent parameters

For each patient, the patient-dependent parameters were collected or calculated in order to investigate the relationship between these parameters and [^18^F]FDG PET image quality. Body weight (BW) [kg] and body height (H) [m] were collected from the patient files. Body mass index (BMI), body weight per body height (BWH) and body surface area (BSA) were calculated as follows [25]:

$${\text{Body}}\;{\text{mass}}\;{\text{index }}\left( {{\text{BMI}}} \right): = \frac{M}{{H^{2} }}({\text{kg}}/{\text{m}}^{{2}} )$$

$${\text{Body}}\;{\text{weight}}\;{\text{per}}\;{\text{bodyheight}}\left( {{\text{BWH}}} \right): = \frac{M}{H}\;({\text{kg}}/{\text{m}})$$

$${\text{Body}}\;{\text{surface}}\;{\text{area }}\left( {{\text{BSA}}} \right): = 0.007184 \times M^{0.425} \times H^{0.725} \;({\text{m}}^{{2}} )$$

### [^18^F]FDG PET/CT

Patients were prepared according to local protocols for [^18^F]FDG PET/CT in children and adults. Children had to fast for four hours prior to injection and were stimulated to drink water (body weight × 10 ml < 10 years or 500–1000 ml > 10 years) during the last two hours before injection. Adults had to fast for six hours and drink 1000 ml of water before injection. Additionally, 17 children and 10 adults followed a carbohydrate-restricted diet for 24 h and fasted the last 12 h to suppress myocardial [^18^F]FDG uptake. Uptake of [^18^F]FDG in brown adipose tissue was suppressed in 91 children and 17 adults by administering Propranolol (0.33 mg [mg] x BW with a maximum of 20 mg one hour before the injection for children and a fixed dose of 20 mg for adults. Dosing of [^18^F]FDG activity was determined with the local linearized quadratic dose regimen of 1.7 megabequerel per kilogram (MBq/kg) (≤ 55 kg, 68 children, with a minimum of 14 MBq; 36 adults); 2.3 MBq/kg (55-70 kg, 26 children; 29 adults); 3.0 MBq/kg (71–95 kg, 7 children; 10 adults); and 4.0 MBq/kg (≥ 96 kg, 1 child; 10 adults) and was intravenously injected (children median: 82 MBq; range 13–392 MBq and adults median: 138 MBq; range 56–532 MBq) followed by resting in a warm and quiet room. PET images were acquired 60 ± 5 min for children and 59 ± 3 min for adults after tracer injection in supine position on a Siemens Biograph mCT PET/CT scanner (Siemens Healthineers, Erlangen, Germany). According to our local scan protocol, first a whole-body low-dose CT with optimized parameters [26–28] was acquired for attenuation correction and localization purposes (Teenager/adult values in brackets). 80 kV (120 kV); Quality reference mAs 140 (40 mAs); automatic exposure control strength strong (average); rotation time 0.5 s; pitch 0.8 mm; slice thickness 2 mm (3 mm); reconstructed slice thickness 2 mm (3 mm); and a Siemens B19f low-dose for emission computed tomography kernel. Directly after the low-dose CT, PET acquisition started with an acquisition time of 3 min per bed position (mbp) from the inguinal region to the skull base with the arms up (61 children and all adults). Another 41 children (31 arms up, 10 arms down) were scanned from the skull base to the feet with 2 mbp for the additional lower extremities. Once acquisition was finished, PET scans were corrected for scatter and attenuation using the low-dose CT, followed by reconstruction using ordered subset expectation maximization (OSEM; 3 iterations; 21 subsets; point spread function (PSF) recovery; time of flight (TOF); a 3 mm Gaussian post-reconstruction filter on a matrix of 200 × 200 with a pixel size of 4.1 × 4.1 mm and 3 mm slice thickness.

### Image analysis

The reconstructed PET scans were analysed for image quality based on the approach as described by de Groot et al. [15] and previously used by Cox et al. [29]. Image quality was measured with the signal-to-noise ratio (SNR) in the liver. To determine the SNR liver, a volume of interest (VOI) was placed in a lesion-free homogeneous part of the right liver lobe (diameter 3 cm) using Hermes Hybrid viewer 2.6D software (Hermes Medical Solutions, Stockholm, Sweden). The VOIs were placed at least 1 cm from the edge of the liver to avoid partial volume effects. The SNR liver was calculated by dividing the liver standard uptake value (SUV) mean normalized for body weight (SUVbw) by the standard deviation (SD) [15]. The liver SUVmean can also be used as a measure for liver deoxyglucose metabolism [30, 31]. In order to compare the liver deoxyglucose metabolism between children and adults, the liver SUVmean normalized by body surface area (SUVbsa) was determined. This value is less dependent on body size and age compared to SUVbw and therefore a better value for liver deoxyglucose metabolism [30, 31].

PET image quality depends on the time per bed position and the amount of administered activity. The product of these parameters is the dose time product (DTP [MBq·min]). The SNR liver can be normalized (SNRnorm liver [(MBq·min)^−1/2^]) for the administered activity and scan time per bed position by assuming Poisson statistics in which noise increases with the square root of the signal [15]. The SNRnorm liver is assumed independent of scan time and administered activity.

1 $${\text{SNRnorm liver}} = \frac{{ {\text{SNR}}\;{\text{ liver}}}}{{\sqrt {{\text{DTP}}} }}$$

### Statistical analysis

All statistical analyses were performed using Graphpad PRISM version 9. In order to test for significant differences in liver deoxyglucose metabolism (liver SUVmean) and image quality (SNR liver and SNRnorm liver) between children and adults, an unpaired t-test (α = 0.05) or a Mann–Whitney U test (α = 0.05) after testing the data for normality by a Shapiro–Wilk test (α = 0.05) was performed. Furthermore, a Pearson product correlation was run to determine the correlations between SNR liver and SNRnorm liver with age in both patient groups.

The coefficient of determination (Pearson *R*^2^) was used to select the best patient-dependent parameter after curve fitting of each parameter with SNRnorm liver. Curve fitting was applied to the parameters by using a power function (Eq. 2) to obtain a nonlinear dosage regimen as described before by the Groot et al. [15]:

2 $${\text{SNR}}_{{\text{norm liver, fit}}} = \alpha p^{ - d} {,}$$

where α and d are fit parameters and p is the patient-dependent parameter.

Curve fitting was also performed with Eq. 3 to obtain a linear dosage regimen:

3 $${\text{SNR}}_{{\text{norm liver, fit}}} = \alpha p^{ - 0.5} {,}$$

where α is the fit parameter and p is the patient-dependent parameter.

Preference between the two models for each patient-dependent parameter was determined by the difference between the Akaike’s corrected information criterion values (ΔAICc) [32].

To determine significant differences between the patient-dependent parameter fit with the highest R^2^ and the other patient-dependent parameters fits, the relative error between SNRnorm liver and SNRfit liver was calculated for each data point using (SNRfit liver – SNRnorm liver)/SNRfit liver × 100%. The relative error between the fits was tested with one-way ANOVA test (α = 0.05) or a nonparametric Friedman test with additional post hoc tests after testing for normality [15].

The fit parameters of the best patient-dependent parameter were used for the dose regimen proposal. The fit parameters were entered in the following expressions:

Combining Eq. 1 and 2 results in the following nonlinear expression for the DTP [MBq·min] [15]:

4 $${\text{DTP}} = \frac{{{\text{SNR}}\;{\text{liver}}^{{2}} }}{{\alpha^{2} }} \times p^{2d} {.}$$

In the linear dosage regimen (d = 0.5), DTP [MBq·min] is indicated by the following linear expression:

5 $${\text{DTP}} = \frac{{{\text{SNR}}\;{\text{liver}}^{2} }}{{\alpha^{2} }} \times p{.}$$

## Results

### Patient-dependent parameters

The measurements and calculations of the paediatric patient-dependent parameters are displayed in Table 1.

**Table 1:** Paediatric patient-dependent parameters and SUV values

Parameters	Mean ± SD	Range	Median
Body weight (kg)	45.9 ± 19.9	3.8–96.0	46.5
Body height (m)	1.49 ± 0.26	0.57–1.86	1.58
BMI (kg/m^2^)	19.4 ± 4.3	11.7–33.9	18.8
BWH (kg/m)	29.6 ± 9.9	6.7–55.2	29.6
BSA (m^2^)	1.5 ± 0.5	0.3–2.5	1.7

### Liver deoxyglucose metabolism

An unpaired t-test was performed to compare the liver deoxyglucose metabolism between children and adults. This showed that SUVbsa in children (0.57 ± 0.10) was significant lower (*t* (185) = 7.82, *p* < 0.001) compared with SUVbsa in adults (0.69 ± 0.10) with a mean difference of 0.11 (95%CI, 0.09 to 0.14).

### Image quality

**Fig. 1:**
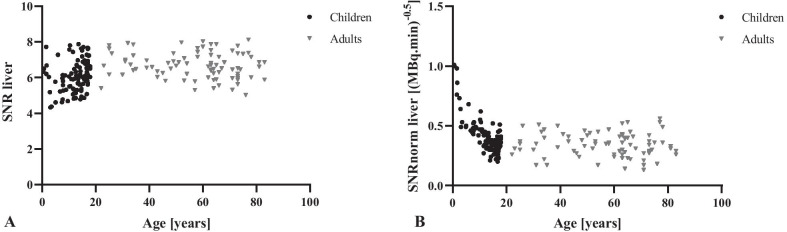
Scatterplots of the SNR liver (**a**) and the SNRnorm liver (**b**) against age

To compare the SNR liver between children and adults an unpaired T-test was performed, which showed a significant (*t* (185) = 4.561, *p* < 0.001) difference in mean SNR liver between children (6.1 ± 0.9) and adults (6.7 ± 0.7). Also, the Mann–Whitney U test to compare the mean SNRnorm liver between adults (0.34 ± 0.10) and children (0.41 ± 0.15) revealed a significant (*U* = 3087, *p* < 0.001) difference between these two groups as displayed in Fig. 1. In children, the correlation between SNR liver and age showed a weak significant positive correlation (*r* = 0.272, *n* = 102, *p* = 0.006). For adults, both SNR liver and SNRnorm liver showed no correlations with age of, respectively, *r* = -0.138, *n* = 85, *p* = 0.208 and *r* = -0.092, *n* = 85, *p* = 0.400. This is in contrast to SNRnorm liver in children, which strongly correlated with age in a negative direction (*r* = -0.795, *n* = 102, *p* < 0.001).

### Curve fitting

**Fig. 2:**
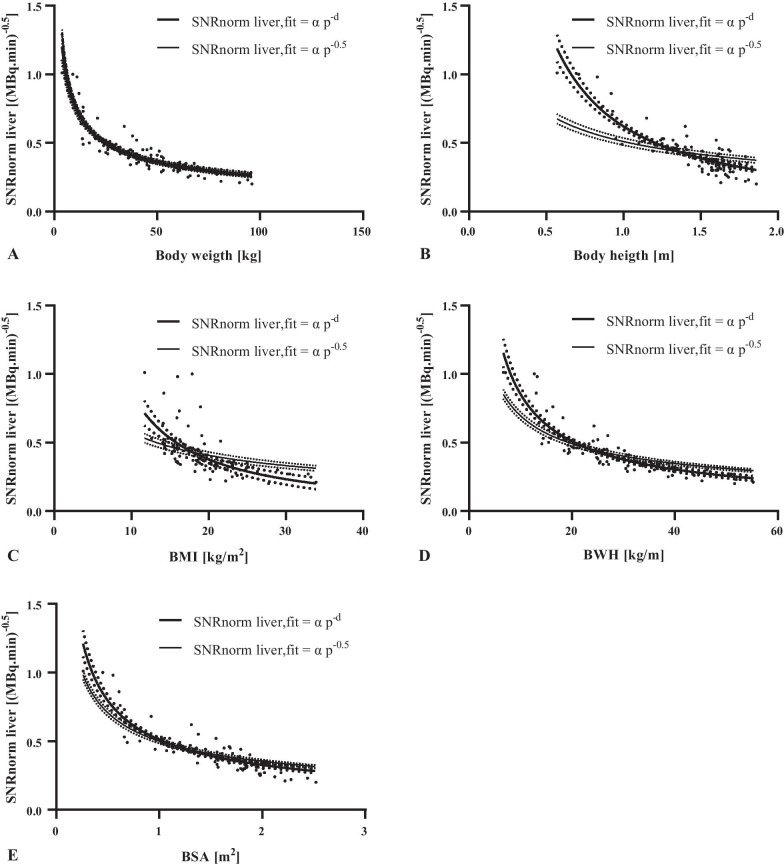
Curve fitting to determine the fit parameters for a linear and nonlinear dosage regimen of the mean SNRnorm liver ([MBq·min)^−1/2^] versus body weight (**a**), body height (**b**), BMI (**c**), BWH (**e**) and BSA (**e**). The dashed lines represent the 95% confidence intervals of the fits

The results of the curve fitting of SNRnorm liver with patient-dependent parameters are presented in Fig. 2 and Table 2. As can be seen body weight shows for both curves the highest R^2^ (0.81 and 0.80, respectively). The nonlinear model is the preferred model, according to the AICc. For this model, the Friedman test with additional Dunn–Bonferroni post hoc test revealed only a significant difference (*p* < 0.05) between the fit of body weight and the fit of body height. (Table 2).

**Table 2:** Fits of SNRnorm liver with the patient-dependent parameters

Patient-dependent parameters	SNRnorm liver, fit = α p^−0.5^			SNRnorm liver, fit = α p^−d^					
	*α*	*R*^2^	*p* value	*α*	*d*	*R*^2^	*p* value	ΔAICc	AIC%
Body weight (kg)	2.51	0.80	-	2.23	0.46	0.81	-	1.86	23.8 vs 71.20
Body height (m)	0.51	0.47	< 0.001	0.62	1.15	0.78	0.046	88.44	< 0.01 vs > 99.99
BMI (kg/m^2^)	1.82	0.27	< 0.001	13.26	1.19	0.41	> 0.999	18.29	0.01 vs 99.99
BWH (kg/m)	2.20	0.67	< 0.001	4.73	0.74	0.78	> 0.999	33.61	< 0.01 vs > 99.99
BSA (m^2^)	0.50	0.75	0.001	0.51	0.64	0.81	0.085	23.89	< 0.01 vs > 99.99

The SNR value for the proposed dosage regimen was set to 6.5. This value was based on an internal adult analysis concerning the shift form a linearized quadratic dosage regimen to a quadratic dosage regimen. This analysis showed that an SNR of 6.5 revealed satisfactory image quality for our nuclear medicine physicians. Thus, children showed a lower mean SNR compared to adults and the nuclear medicine physicians experienced sometimes scans with a poor image quality in children < 20 kg. Therefore, three nuclear medicine physicians decided to equalize these values and use an SNR of 6.5 for both dosage regimens.

The proposed dedicated dosage regimen was obtained by combining Eq. 4 with an SNR liver of 6.5 and the fit parameters of body mass which are α = 2.23 (95% CI 1.90 to 2.51) and d = 0.46 (95% CI 0.43 to 0.50). This resulted in the following nonlinear expression for the DTP [MBq·min] with a minimum of 26 MBq conform the guidelines [7]:

6 $${\text{DTP}} = 8.5 \times M^{0.92} {.}$$

### Comparison of the proposed dosage regimen with the adult dosage regimen and the current paediatric dosage regimen in guidelines.

**Fig. 3:**
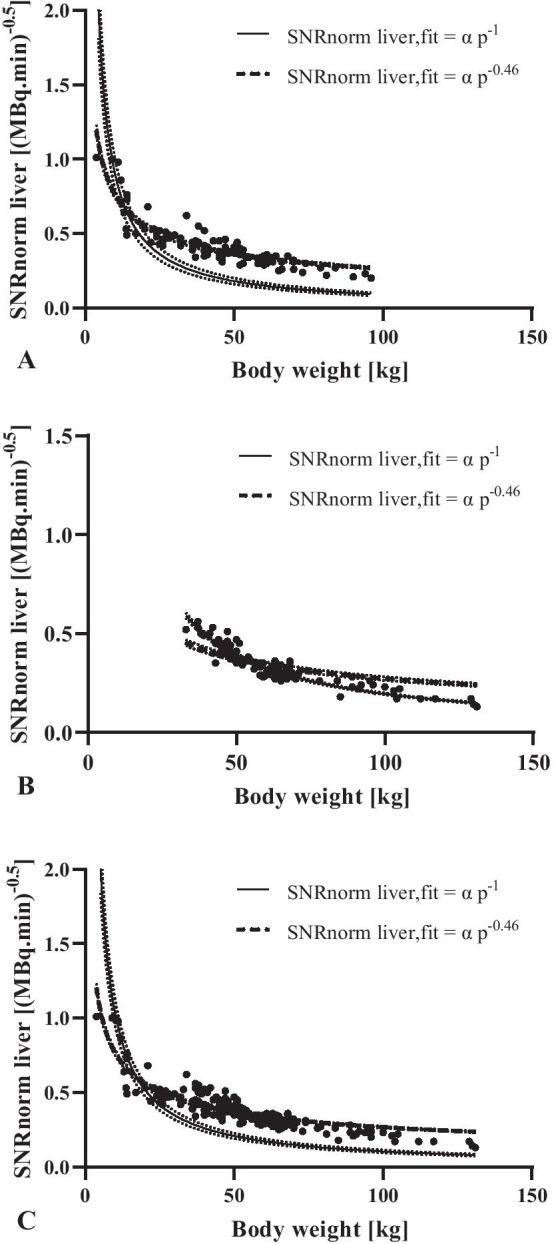
Comparison between SNRnorm liver fits that corresponds with the proposed nonlinear dosage regimen (parameter d fixed to 0.46) and a quadratic dosage regimen (parameter d fixed to 1) for children (**a**), adults (**b**) and both groups (**c**). The dashed lines represent the 95% confidence intervals of the fits

Figure 3 shows the fits of SNRnorm liver versus body weight that corresponds to the proposed nonlinear paediatric dosage regimen and the new Erasmus MC quadratic dosage regimen for adults. It can be seen that the nonlinear dosage regimen fits perfectly for children (Fig. 3a) and not for adults (Fig. 3b), whereas a quadratic dosage regimen fits perfectly for adults and not for children. No universal dose regimen could be found for children and adults together neither nonlinear nor quadratic (Fig. 3c).

**Fig. 4:**
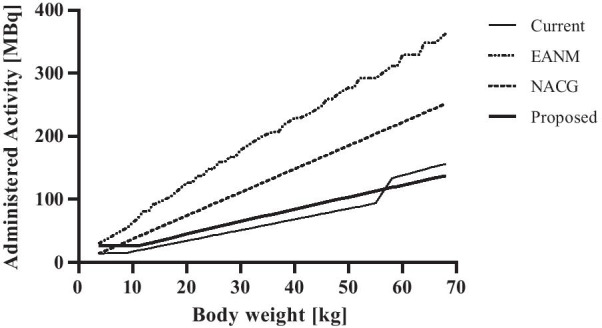
Comparison of [^18^F]FDG dosage regimens. Current study, EANM, NACG and the proposed dose regimen

In Fig. 4, our dosage regimens with an acquisition time of 3 mbp are compared with those of the EANM and NACG guidelines. As can be seen our new dosage regimen (65 MBq and 3.6 mSv [33]) will reduce the amount of administered activity and radiation dose with 41% (NACG: 111 MBq and 6.2 mSv) and 63% (EANM: 178 MBq and 9.9 mSv) for a child of 30 kg, despite the higher amount of injected activity compared to the dosage regimen used in the current study (51 MBq and 2.9 mSv).

## Discussion

In this study, the relation between patient-dependent parameters and [^18^F]FDG PET image quality was investigated in order to propose a dedicated paediatric dose regimen that aims for constant and sufficient image quality. To the best of our knowledge, this study is the first that investigates the relationship between paediatric patient-dependent parameters and [^18^F]FDG PET image quality based on SNR. This study has demonstrated that body weight is the parameter with the greatest effect on [^18^F]FDG image quality in children. Another important finding was that SNRnorm values were significant higher in children and correlated more strongly with age than in adults. Furthermore, this is the first study providing insight into the differences between paediatric and adult dosage regimens. This insight emphasizes the need for a dedicated paediatric dosage regimen, especially for young children.

Liver deoxyglucose metabolism in children was shown to be significantly lower than in adults. These results confirm the results obtained in a paediatric study by Yeung et al.[30]. The increase in [^18^F]FDG uptake during growth may be caused by age related changes in liver volume [34] and function of hepatocytes [35, 36]. Furthermore, the significant changes in body size, body composition [34, 37] and blood volume during growth [37] could also account for an increase in [^18^F]FDG uptake. Not only body size and age affected SUV measurements but also differences in uptake period, plasma glucose, recovery coefficient and partial volume artefacts [38]. The contribution of these factors may be limited in this study due to the use of standardized protocols.

Despite lower SUVbsa mean values and concomitant lower liver [^18^F]FDG uptake, small children showed high SNRnorm values, which implies that less activity is needed to obtain sufficient image quality (Fig. 1a). This is probably caused by less attenuation and scatter due to the smaller body sizes. In contrast to SNRnorm, SNR has almost no correlation with age (Fig. 1b). This means that, despite the quite large spread (range 4.3–8.1), the currently used dosage regimen already provides a constant image quality throughout our patient population.

Body weight was the patient-dependent parameter with the highest coefficient of determination for both fit models for SNRnorm. The fit parameters (α = 2.23 and d = 0.46) of the preferred model are in line with the fit parameters (α = 3.2 and d = 0.52) obtained by van Gent et al. [21] in a paediatric pilot study of 20 patients. The fit of the preferred model was not significantly different from the fits of BMI, BWH and BSA, even though body mass was used to define the optimal dose regimen as it is more practical than BMI, BWH and BSA. Although the selected model explains 81% of variability in SNRnorm among patients, the remaining 19% was not explained. This variability might be caused by differences in patient size, not covered by body weight. Unknown inhomogeneities within the liver VOIs might also cause variability of SNRnorm. Nevertheless, our results are consistent with earlier adult [^18^F]FDG studies of de Groot et al.[15] and Menezes et al.[39]. These studies determined body weight with the highest coefficient of determination with SNRnorm with, respectively, R^2^’s of 0.77 (OSEM 3D + PSF + TOF) and 0.86 (OSEM 3D + PSF) using comparable scanners to ours. Body weight was also identified as the best single predictor for image quality by Accorsi et al. [10] at a comparable R^2^ of 0.86 using a different camera, reconstruction method and the noise equivalent count rate density (NECRD) as measure of image quality. The NECRD is derived from the noise equivalent count rate (NECR) method [19], which is considered to be more objectively related to SNR, since it is not affected by possible differences in liver metabolism and reconstruction methods [40–42]. However, at that moment NECRD was not validated with a visual assessment, and therefore, the estimated sufficient image quality and proposed dosage regimen could be unreliable [43].

In contrast to the studies mentioned above, this study directly compared the models of children and adults. It was not possible to find a universal dosing regimen linking paediatric and adult protocols (Fig. 3). This insight emphasizes the need for a dedicated paediatric dosage regimen, especially for young children. These results are in agreement with the EANM adult guidelines [14], which recommends using a quadratic dosage regimen, especially for patients > 75 kg, as this compensates for the lower image quality caused by substantial attenuation when using a linear dosage regimen [15]. In addition, they recommend that the linear dosage regimen is appropriate to use for patients < 75 kg, but this is not supported by data or references.

Our proposed paediatric [^18^F]FDG dosage regimen (acquisition time 3 mbp) showed a reduction of the amount of administered activity and effective dose of 41% (NACG) and 63% (EANM) in a child of 30 kg (Fig. 4). Our findings broadly support the work of other studies in this area, which also found reductions of 40–50% [10, 11, 22, 23], although they used other dosage regimens, scanners and reconstruction methods.

A limitation of our study is the absence of raw data due to the retrospective approach. Therefore, NECR analysis of the raw data to support the SNR data was not possible. The NECR data are more objective since they are not affected by differences in liver deoxyglucose metabolism and reconstruction parameters. An earlier adult study of Menezes et al. [39] determined that both SNR and NECR showed the best correlation with body weight. The clinical SNR analysis could be replicated in a phantom study by de Groot et al. [15] and this showed that the effects of liver glucose metabolism and reconstruction parameters are either rare or not as influential compared to attenuation effects. Another limitation is that we included only 85 adult patients against 102 children. However, we tried to create as much as possible equal patient distribution and adult patients below 55 kg and children above 95 kg are rare in our patient population. Furthermore, it should be pointed out that our study has been primary concerned with optimizing radiation dose from [^18^F]FDG rather than from both [^18^F]FDG and CT. The low-dose CT protocols of our scanners have already been optimized to reduce radiation dose (Child of 30 kg: DLP ± 75 mGy∙cm and 1.4 mSv [44]) and to maintain sufficient image quality as with phantom studies in the past [26–28].

The results of this study are only valid for Siemens Biograph mCT scanners with an OSEM 3D + PSF + TOF reconstruction. However, the objective method applied in this study can be used for further investigation for other scanners and reconstruction methods to obtain evidence-based recommendations for different types of scanners in the guidelines. Recently, three paediatric PET/magnetic resonance imaging (PET/MR) studies concerning the newest generation large field of view PET scanners with solid-state silicon photomultipliers and higher TOF resolution showed already that even more dose reduction is possible due to their higher sensitivity [22, 45, 46], especially when MR replaces CT for localization and attenuation correction [47]. Another promising tool is the new block sequential regularized expectation maximization (BSREM/Q.Clear) reconstruction software [48–50] that generates images with higher image quality, which allows dose reduction. In the future, another step in dose reduction will be taken with the implementation of artificial intelligence (AI) technology in nuclear medicine imaging. AI technology offers a wide range of application opportunities for low-dose PET scans for example AI imaging [51, 52], AI reconstruction [53] and AI post-reconstruction image enhancement [54].

## Conclusion

Body weight has the greatest effect on [^18^F]FDG PET image quality in children. The proposed nonlinear dosage regimen based on body mass will provide a constant and clinical sufficient image quality with a reasonable radiation dose and present a significant reduction of the administered activity compared to the current guidelines. A dedicated paediatric dosage regimen is necessary, as a universal dosing regimen for paediatric and adult is not feasible.

## Data Availability

The data supporting our findings are available upon request.

## Abbreviations

^18^F-FDG: ^18^Fluorine-fluoro-2-deoxyglucose
PET/CT: Positron emission tomography/computed tomography
SNR: Signal-to-noise ratio
SNRnorm: Normalized signal-to-noise ratio
ALARA: As low as reasonably achievable
SNMMI: Society of Nuclear Medicine and Molecular Imaging
EANM: European Association of Nuclear medicine
NACG: North American Consensus guidelines
BW: Body weight
BMI: Body mass index
BWH: Body weight per body height
BSA: Body surface area
MBq/kg: Megabequerel per kilogram
OSEM: Ordered subset expectation maximization
PSF: Point spread function
TOF: Time of flight
VOI: Volume of interest
SD: Standard deviation
DTP: Dose–time product
AICc: Akaike’s corrected information criterion values
ANOVA: Analysis of variance
NECRD: Noise equivalent count rate density
NECR: Noise equivalent count rate
PET/MR: Positron emission tomography/magnetic resonance imaging
BSREM: Block sequential regularized expectation maximization
AI: Artificial intelligence
